# Development of a national maternity early warning score: centile based score development and Delphi informed escalation pathways

**DOI:** 10.1136/bmjmed-2023-000748

**Published:** 2024-05-15

**Authors:** Stephen Gerry, Jonathan Bedford, Oliver C Redfern, Hannah Rutter, Mae Chester-Jones, Marian Knight, Tony Kelly, Peter J Watkinson

**Affiliations:** 1 Nuffield Department of Orthopaedics, Rheumatology, and Musculoskeletal Sciences, University of Oxford, Oxford, UK; 2 University of Oxford Nuffield Department of Clinical Neurosciences, Oxford, UK; 3 Milton Keynes University Hospital NHS Foundation Trust, Milton Keynes, UK; 4 Maternity and Neonatal Safety Improvement Programme, NHS England, London, UK; 5 National Perinatal Epidemiology Unit, University of Oxford, Oxford, UK; 6 National Institute for Health and Care Research (NIHR) Oxford Biomedical Research Centre, Oxford, UK

**Keywords:** Critical care, Obstetrics, Pregnancy complications

## Abstract

**Objective:**

To derive a new maternity early warning score (MEWS) from prospectively collected data on maternity vital signs and to design clinical response pathways with a Delphi consensus exercise.

**Design:**

Centile based score development and Delphi informed escalation pathways.

**Setting:**

Pregnancy Physiology Pattern Prediction (4P) prospective UK cohort study, 1 August 2012 to 28 December 2016.

**Participants:**

Pregnant people from the 4P study, recruited before 20 weeks' gestation at three UK maternity centres (Oxford, Newcastle, and London). 841, 998, and 889 women provided data in the early antenatal, antenatal, and postnatal periods.

**Main outcome measures:**

Development of a new national MEWS, assigning numerical weights to measurements in the lower and upper extremes of distributions of individual vital signs from the 4P prospective cohort study. Comparison of escalation rates of the new national MEWS with the Scottish and Irish MEWS systems from 18 to 40 weeks' gestation. Delphi consensus exercise to agree clinical responses to raised scores.

**Results:**

A new national MEWS was developed by assigning numerical weights to measurements in the lower and upper extremes (5%, 1%) of distributions of vital signs, except for oxygen saturation where lower centiles (10%, 2%) were used. For the new national MEWS, in a healthy population, 56% of observation sets resulted in a total score of 0 points, 26% a score of 1 point, 12% a score of 2 points, and 18% a score of ≥2 points (escalation of care is triggered at a total score of ≥2 points). Corresponding values for the Irish MEWS were 37%, 25%, 22%, and 38%, respectively; and for the Scottish MEWS, 50%, 18%, 21%, and 32%, respectively. All three MEWS were similar at the beginning of pregnancy, averaging 0.7-0.9 points. The new national MEWS had a lower mean score for the rest of pregnancy, with the mean score broadly constant (0.6-0.8 points). The new national MEWS had an even distribution of healthy population alerts across the antenatal period. In the postnatal period, heart rate threshold values were adjusted to align with postnatal changes. The centile based score derivation approach meant that each vital sign component in the new national MEWS had a similar alert rate. Suggested clinical responses to different MEWS values were agreed by consensus of an independent expert panel.

**Conclusions:**

The centile based MEWS alerted escalation of care evenly across the antenatal period in a healthy population, while reducing alerts in healthy women compared with other MEWS systems. How well the tool predicted adverse outcomes, however, was not assessed and therefore external validation studies in large datasets are needed. Unlike other MEWS systems, the new national MEWS was developed with prospectively collected data on vital signs and used a systematic, expert informed process to design an associated escalation protocol.

**WHAT IS ALREADY KNOWN ON THIS TOPIC**

- Maternal early warning scores (MEWS) are widely used to help identify physiological deterioration during pregnancy
- Most MEWS were not developed based on evidence based research

**WHAT THIS STUDY ADDS**

- A new national MEWS was developed, based on the results of a large prospective study
- Compared with other commonly used MEWS, the new national MEWS showed a more manageable alert rate in a healthy population
- How well the tool predicts adverse outcomes, however, was not assessed

**HOW THIS STUDY MIGHT AFFECT RESEARCH, PRACTICE, OR POLICY**

- The new national MEWS is programmed for implementation across the English NHS
- The effects of consistency, a reduced alert rate in a healthy population, and Delphi based escalation protocol will be monitored
- The MEWS and escalation pathways could be translated to other healthcare systems with a few modifications

## Introduction

Maternal early warning scores (MEWS) are used extensively in hospitals in the UK and internationally by midwives and doctors to monitor the physiology of the pregnant women, identify signs of clinical deterioration, and potentially prevent morbidity or mortality.^1 2^ Typically, MEWS are based on a scoring system where numerical weights are allocated to each measured vital sign, with the weight reflecting how extreme the vital sign is thought to be. These values are summed to make a total score.^2^ Alternatively, MEWS use a colour coding system, where combinations of colours are used to indicate severity.^3^ Actions might be required if a patient's total score, or combination of colours, reaches a specific threshold value. The parameters included might vary between MEWS, as well as the threshold values for points scored. No consensus exists about which score works best, and many different scores are used in clinical practice across the NHS and internationally.^2^ Most existing scores have been developed based on clinical consensus, rather than evidence.^4–7^ A standardised MEWS, supported by empirical data, is needed.

In the Pregnancy Physiology Pattern Prediction (4P) prospective cohort study, 1041 women took part, and vital signs were measured throughout the antenatal period and for two weeks postnatally.^8^ This article uses the term "woman" throughout and includes all female people, including those who do not see themselves as women. The primary objective of this study was to develop a database of measurements of vital signs during pregnancy, labour, and the postpartum period from which estimates of population distributions and associated centiles could be derived. The secondary objective was to use this information to develop a centile based MEWS system.^9^ We have previously shown how early warning scores can be derived from distributions of vital signs.^10^

The different MEWS used in the UK causes substantial variation in practice, with little evidence of effective implementation.^11 12^ Appropriate escalation pathways in response to raised early warning scores are essential to ensure detection reduces avoidable morbidity and mortality. A Delphi process is an established method to develop expert consensus.^13^ The key principles of the Delphi method are that group consensus is more valid than individual opinions, and that structured communication is an effective way to deal with complex problems.^14^ Delphi processes have previously been used to inform the development of non-maternity early warning scores.^15 16^

In this study, we used data from the 4P study and a Delphi consensus exercise to develop an empirically derived, expert informed MEWS tool to identify and respond to deterioration during pregnancy. The performance of the model could not be assessed because of the small number of people with poor outcomes in our dataset, and therefore triggering rates were assessed only in a healthy population.

## Methods

We conducted a mixed methods study based on multicentre, observational data from the 4P study to develop a new national MEWS. A Delphi process was used to design a consensus derived escalation protocol.

### Development of a new national MEWS

The aim of the study was to derive a score that was similar in appearance and functionality to existing MEWS, but with an evidence based approach to determine the threshold values. Most existing MEWS allocate 0, 1, or 2 points, separately, to each of six vital signs, according to the degree of abnormality (ie, a normal value would score 0 points and extremely abnormal values would score 2 points). The scores are then summed to calculate a total score, often referred to as MEWS. Although the accuracy of the score could be improved by adjusting some of these design decisions, we chose to retain these elements to allow for ease of use and implementation, and so that the score would be familiar to clinicians; these scores are often calculated by hand on paper charts.

#### Participants

We used vital signs collected in the 4P study during the antenatal period and two weeks post partum to derive a new national MEWS, excluding the period around labour. 4P was a longitudinal cohort study where pregnant women were approached for recruitment before 20 weeks of pregnancy at four UK maternity centres. We included women aged ≥16 years, with a singleton pregnancy, and ASA I (category I of the American Society of Anesthesiologists' classification of physical status before pregnancy as a normal healthy patient without any clinically important comorbidity and without clinically significant past or present medical history). Therefore, the estimated centiles represent normal ranges in a healthy population. Although the inclusion criteria represented a relatively healthy cohort, we did not remove women from the dataset for any new medical complications that arose, to mimic routine practice. Women were prospectively recruited from three UK sites during the period 1 August 2012 to 28 December 2016. Full details are described elsewhere.^8 17^

#### Variables, data sources, and measurement

We collected vital signs at clinic visits every 4-6 weeks: blood pressure, heart rate (pulse rate), oxygen saturation, temperature, and respiratory rate. Further details of our standard operating procedure and measurement equipment are described elsewhere.^9^ We also collected personal information at the initial assessment (age, height, weight, self-reported ethnic group, number of previous pregnancies, and smoking status), past medical and obstetric history (from participants' notes), current health status, pregnancy related health, and current drug treatments.

#### Study size

A priori sample size calculations are described in previous publications.^8 9^ In brief, to create an evidence based early warning score, we wanted a 95% confidence interval (CI) with a standard error of <0.10×standard deviation (SD) at the boundaries. We estimated a sample size of 1000 women would achieve a standard error of 0.05×SD at the 2.5th and 97.5th centiles, and even greater precision at the less extreme centiles. Adequate precision was also met for any subgroup analysis; for example, we estimated a sample size of 300 women would achieve a standard error of 0.1×SD at the 2.5th and 97.5th centiles.

#### Quantitative variables and statistical methods

We previously calculated models of distributions of individual vital signs for the antenatal and postpartum periods.^8 17^ This method allowed us to calculate and plot smooth centiles according to the stage of pregnancy. These univariable models were fitted with the generalised additive models for location, scale, and shape,^18^ which allows for different families of statistical distributions to be used (eg, the Box-Cox Cole and Green distribution).^8 17^ Also, different smoothing functions were available to smooth across the explanatory variable (gestational age or days after delivery), such as P splines or fractional polynomials. The best fitting model for each vital sign was chosen by inspecting empirical centiles versus fitted centiles, and using model fit criteria, such as the bayesian information criterion.

Threshold values for the new national MEWS were derived from the centiles of each distribution at 34 weeks' gestation (ie, the midpoint of the third trimester), chosen to reflect the use of MEWS in the peripartum period. We used values corresponding to the first, fifth, 95th, and 99th centiles to define threshold values for each increase in score (0, 1, and 2 points), consistent with our previous centile based early warning scores.^10^ For example, a score of 2 points would be given to each vital sign that was either <1st centile or >99th centile. For oxygen saturation, we only assigned weightings to low values, specifically the second and 10th centiles.^10^ The total MEWS value was calculated by summing individual points for vital signs, ranging from 0 to 12.

Because the 4P cohort comprised a relatively low risk population with few adverse outcomes (online supplemental table 3), evaluating the performance of the new national MEWS by calculating discrimination and calibration metrics (as is the conventional approach for prognostic model evaluation) was not possible.^19^ Instead, we assessed performance in the cohort descriptively by looking at triggering rates in a healthy population.

10.1136/bmjmed-2023-000748.supp1 Supplementary data

We compared the distributions of total scores for the new national MEWS with all observations taken between 18 and 40 weeks' gestation with two comparable systems developed in Ireland and Scotland.^3^ ^20^ These two systems were chosen as representing national maternal early warning systems in current use. We also included the national early warning score (NEWS2) that was of secondary interest in the main antenatal period, but of greater interest in the early antenatal and postnatal periods. The Scottish and Irish MEWS are similar in design to the new national MEWS (≤2 points for each vital sign, triggering escalation of care at a total score of ≥2 points). The NEWS2 can score up to 3 points for each vital sign, and typically triggers at 5 points. We only used observation sets where all vital signs were recorded simultaneously (complete case analysis). The distribution of total scores was plotted for each of the three MEWS by considering all observation sets in the cohort. This approach included multiple observation sets for each person, and therefore the distribution of each person's highest MEWS was also plotted. The distribution of individual scores for vital signs was also plotted separately. We explored the relation between mean total MEWS and gestational age, applying a locally weighted smoothing smoother, along with 95% CIs.

#### Early antenatal and postnatal adjustments

Because of the known trends in vital signs in the antenatal and postnatal periods, we investigated whether any adjustments were needed to the new national MEWS in the early antenatal (<18 weeks' gestation) and postnatal (0-16 days after delivery) periods.^5 8 17^ We recalculated score threshold values for each vital sign, with centiles specific to those periods at 16 weeks and at five days, for the early antenatal and postnatal periods, respectively. We then evaluated the performance of the new national MEWS in those early antenatal and postnatal periods. We also compared alerting rates in these periods with NEWS2,^21^ because this scoring system might be considered an appropriate alternative in these contexts. The Scottish and Irish MEWS were also included for completeness.

### Delphi consensus exercise

We undertook a two round Delphi exercise to design a consensus derived escalation protocol of the most appropriate responses to different MEWS values. We identified participants through the Each Baby Counts Learn and Support programme.^22^ We asked the stakeholders involved in the programme to identify a multiprofessional selection of independent colleagues in their respective institutions. Although 5-15 experts are often considered adequate to validate content,^23^ we aimed for responses from >30 participants. We developed a range of clinical scenarios covering possible MEWS values, which were shown to the Delphi participants. The scenarios were presented with parameters for vital signs and associated total MEWS scores. The scoring threshold values were also presented for reference with each scenario.

#### Delphi round 1

We asked the Delphi participants to indicate the escalation responses they felt should occur in response to each set of vital signs and associated MEWS scores. For each clinical scenario, participants were asked to select:

1. The most appropriate primary escalation contact or contacts. Options included: no escalation, midwife, specialty trainee with ≤2 years of obstetric training, specialty trainee with ≥3 years of obstetric training, consultant, critical care outreach, and obstetric emergency call. (Primary escalation contacts were described in line with the current UK system.)
2. The most appropriate action for the frequency of observations. Options included: no change, repeat in one hour, repeat in 30 minutes, repeat in 15 minutes, and move to continuous observations.
3. The most appropriate secondary escalation contact (should the primary contact or contacts fail to attend in the time required). Options were the same as in (1).

#### Delphi round 2

We collated the responses from round 1. Only those participants who responded to round 1 were invited to complete round 2. Participants were asked again to indicate the escalation responses they felt should occur in response to the same scenarios provided in round 1. For round 2, however, each scenario was displayed with the percentage of participants that voted for each response option in round 1. The consensus response was selected where a clinical response to a specific MEWS was selected by ≥50% of respondents.

### Ratification by stakeholders

The suggested responses produced from the Delphi consensus exercise were reviewed and ratified by the core design group, comprising key stakeholders (representation from the relevant royal colleges, specialist societies, and academics with specific interest and experience in this area).

### Patient and public involvement

Patients and/or the public were not involved in the design, or conduct, or reporting of this research, since the study was designed more than a decade ago. The results of the study will, nevertheless, be disseminated to the public and health professionals by press releases and presentations.

## Results

Table 1 shows the baseline characteristics of participants included in the performance assessment, by period of pregnancy. The 4P study included 1041 women in total. In the early antenatal, antenatal, and postnatal periods, 841, 998 and 889 women provided data included in the analysis (online supplemental figure 1). Online supplemental table 3 shows details of pregnancy outcomes and perinatal events. Adverse outcomes were rare.

**Table 1:** Baseline maternal characteristics according to period of pregnancy for data used to evaluate performance of new national maternal early warning score

Characteristics	Early antenatal (<18 weeks' gestation, n=841)	Antenatal (18-40 weeks' gestation, n=998)	Postnatal (0-16 days after delivery, n=889)
Median (range) No of observations for each participant	1 (1-2)	4 (1-7)	10 (1-18)
Mean (SD) age (years)	31.6 (4.67)	31.6 (4.77)	31.7 (4.73)
Mean (SD) weight (kg)	68.1 (13.4)	68.3 (13.6)	67.9 (13.4)
Mean (SD) body mass index	24.9 (4.55)	24.9 (4.61)	24.8 (4.46)
Body mass index category			
Normal weight (18.5-24.9)	489 (58.0)	585 (58.5)	534 (60.1)
Overweight (25.0-29.9)	236 (28.1)	271 (27.2)	233 (26.2)
Obese (≥30)	115 (13.7)	140 (14.0)	118 (13.3)
Nulliparous	366 (43.5)	447 (44.8)	401 (45.1)
Ethnic group			
White	722 (85.9)	853 (85.5)	764 (85.9)
Asian	42 (5.0)	50 (5.0)	39 (4.4)
African or Caribbean	19 (2.3)	21 (2.1)	16 (1.8)
Mixed	16 (1.9)	17 (1.7)	16 (1.8)
Other	42 (5.0)	57 (5.7)	54 (6.1)
Site			
Oxford	661 (78.6)	749 (75.1)	669 (75.3)
Newcastle	103 (12.2)	148 (14.8)	134 (15.1)
London	77 (9.2)	101 (10.1)	86 (9.7)
Smoker	60 (7.1)	69 (6.9)	54 (6.1)
Anaemia	6 (0.7)	7 (0.7)	6 (0.7)
Pregestational diabetes	6 (0.7)	6 (0.6)	6 (0.7)
Pre-existing hypertension†	11 (1.3)	16 (1.6)	14 (1.6)
Cardiac disease‡	12 (1.4)	12 (1.2)	11 (1.2)
Pre-existing renal disease	21 (2.5)	24 (2.4)	18 (2.0)

Data are number (%) unless indicated otherwise.

*Haemoglobin concentration <110 g/L.

†Not receiving drug treatments.

‡Non-ischaemic, non-congenital.

SD, standard deviation.

### New national MEWS

**Figure 1:**
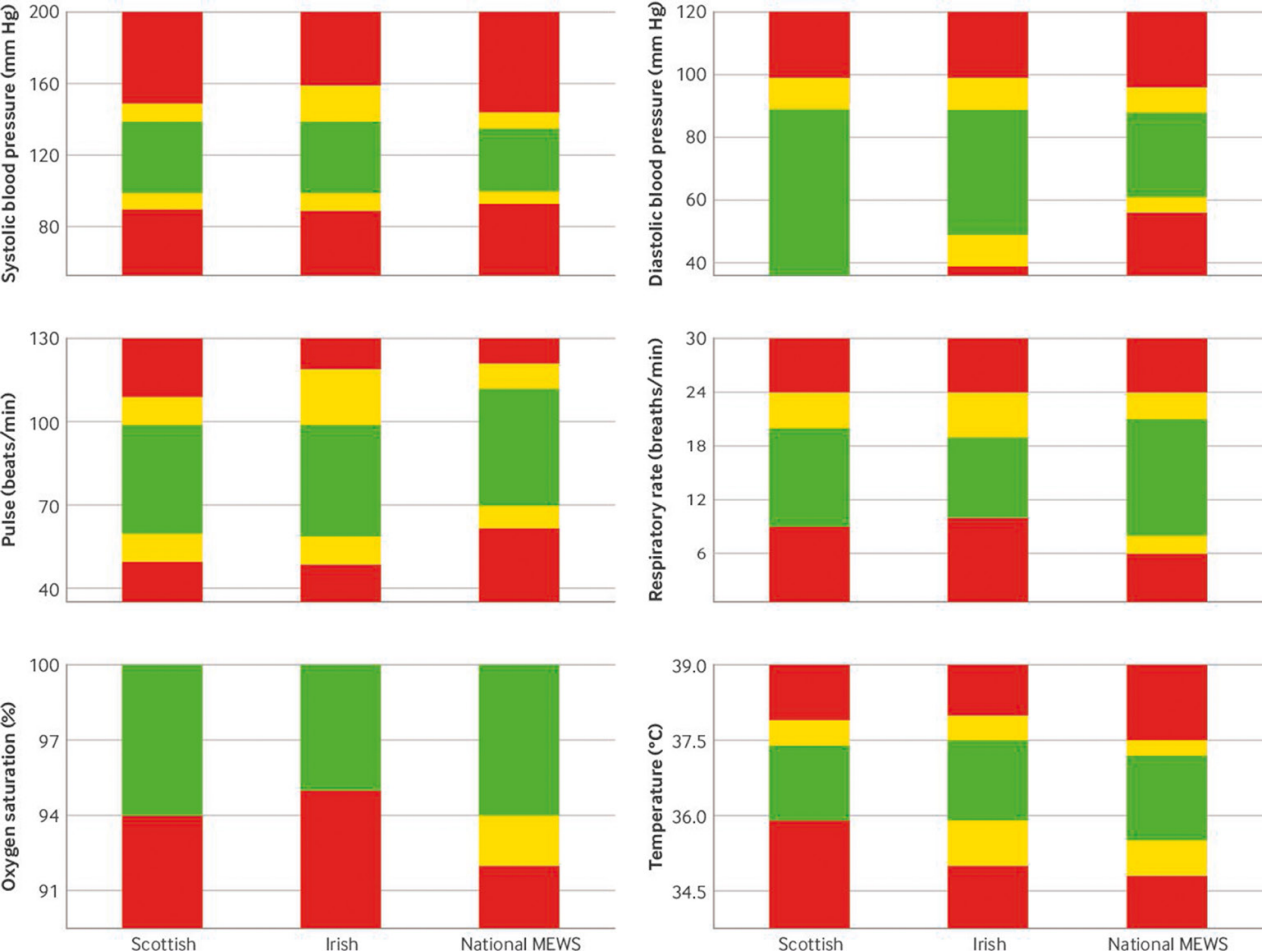
Scoring threshold values for new national maternity early warning score (MEWS), Scottish MEWS, and Irish MEWS, for each vital sign. Green areas score 0 points, yellow areas score 1 point, and red areas score 2 points

Table 2 shows the scoring rules for the new national MEWS. Figure 1 shows a visual comparison of the scoring threshold values with the Scottish and Irish MEWS (and including NEWS2, online supplemental figure 2). The new national MEWS had similar threshold values for systolic blood pressure and respiratory rate but differed for pulse rate and peripheral oxygen saturation (SpO_2_). For some vital signs, the range considered normal and therefore scored 0 points was smaller for the new national MEWS than the Scottish and Irish MEWS (ie, systolic blood pressure and diastolic blood pressure). For other vital signs, the normal range was larger for the new national MEWS (ie, respiratory rate and SpO2). For the remaining vital signs (ie, pulse rate and temperature), the normal range was similar in size to the Scottish and Irish MEWS but was shifted.

**Table 2:** Scoring threshold values of new national maternity early warning score (MEWS) for each vital sign

Vital signs	Score				
	2 points	1 point	0 points	1 points	2 points
Respiratory rate (breaths/min)	≤6	7-8	9-21	22-24	≥25
Oxygen saturation (%)	≤92	93-94	≥95	—	—
Temperature (°C)	≤34.8	34.9-35.5	35.6-37.2	37.3-37.5	≥37.6
Pulse rate (beats/min)	≤62	63-70	71-112	113-121	≥122
Systolic blood pressure (mm Hg)	≤93	94-100	101-135	136-144	≥145
Diastolic blood pressure (mm Hg)	≤56	57-61	62-88	89-96	≥97

Each vital sign was scored independently, and was scored 0, 1, or 2 points. Non-zero (ie, 1 or 2) points can be scored for vital signs that are either high or low, except for oxygen saturation, which only scored for low values (therefore, all values ≥95% scored 0 points). Individual points were summed to calculate a total early warning score. Because each vital sign can score between 0 and 2 points, the total score can range from 0 to 12.

Example: if a woman's respiratory rate was 22 breaths/min, oxygen saturation 96%, temperature 34.7°C, pulse rate 116 beats/min, systolic blood pressure 139 mm Hg, and diastolic blood pressure 102 mm Hg, she would score 1 point for respiratory rate (22-24 range), 0 points for oxygen saturation (≥95% range), 1 point for temperature (34.9-35.5 range), 1 point for pulse rate (113-121 range), 1 point for systolic blood pressure (136-144 range), and 2 points for diastolic blood pressure (≥97 range). The total (MEWS) score would be 6 points (1+0+1+1+1+2).

### Performance of new national MEWS, Irish MEWS, and Scottish MEWS

#### Comparison of predicted alerting rates

**Figure 2:**
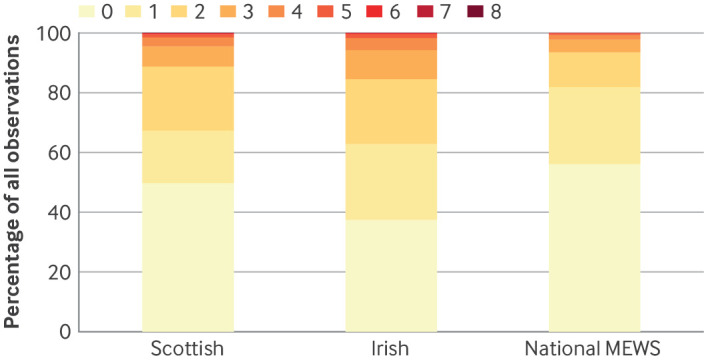
Distribution of total early warning scores for new national maternity early warning score (MEWS), Scottish MEWS, and Irish MEWS, in all observation sets

Figure 2 shows the distribution of total scores for the three MEWS (and including NEWS2, online supplemental figure 3). For the new national MEWS, 56% of observation sets resulted in a score of 0 points, 26% a score of 1 point, 12% a score of 2 points, and 18% a score of ≥2 points. For the Irish MEWS, 37% of observations sets scored 0 points, 25% scored 1 point, 22% scored 2 points, and 38% scored ≥2 points. For the Scottish MEWS, 50% of observations sets scored 0 points, 18% scored 1 point, 21% scored 2 points, and 32% scored ≥2 points.

Online supplemental figure 4 shows the distribution of women's highest MEWS during their pregnancy. For the new national MEWS, the highest score was 0 points for 40% of women, 1 point for 30% of women, 2 points for 18% of women, and ≥2 points for 30% of women; corresponding values for the Irish MEWS were 16%, 23%, 30%, and 61%, and for the Scottish MEWS, 31%, 15%, 33%, and 54%.

Online supplemental figures 5-10 show the scoring for individual vital signs for each MEWS. The new national MEWS had a greater proportion of 1 or 2 point scores than the Irish and Scottish MEWS for systolic blood pressure (new national MEWS 12%, Irish MEWS 8%, and Scottish MEWS 8%) and diastolic blood pressure (new national MEWS 12%, Irish MEWS 3%, and Scottish MEWS 3%). The new national MEWS had fewer 1 and 2 point scores for pulse rate (new national MEWS 10%, Irish MEWS 21%, and Scottish MEWS 21%), respiratory rate (new national MEWS 10%, Irish MEWS 28%, and Scottish MEWS 13%), SpO2 (new national MEWS 5%, Irish MEWS 16%, and Scottish MEWS 5%), and temperature (new national MEWS 11%, Irish MEWS 16%, and Scottish MEWS 16%).

The new national MEWS allocated points (1 or 2) to about 10% of observations for each vital sign, except for SpO2 (5%, a function of scoring only low oxygen saturation values). With the Irish and Scottish MEWS, however, the proportion of observations which scored points varied considerably between vital signs. For example, with both the Irish and Scottish MEWS, only 3% of diastolic blood pressure observations scored, whereas a large proportion of pulse rate (both 21%) and respiratory rate (Irish MEWS 28% and Scottish MEWS 13%) observations scored.

#### Effect of gestational age on MEWS

Online supplemental figure 11 shows the relation between gestational age and total MEWS score. For each of the three MEWS, mean total score was plotted throughout pregnancy. All three MEWS seemed to be broadly similar at the beginning of pregnancy, averaging 0.7-0.9 points, but then differed. The new national MEWS had a lower mean score for the rest of pregnancy, with the mean score broadly constant (0.6-0.8 points). The Irish and Scottish scores increased during the first two trimesters, both averaging >1, before plateauing in the third trimester, with the Scottish MEWS consistently scoring higher than the Irish MEWS. The same trends were seen for the proportion of total MEWS in healthy women that triggered escalation of care (ie, score ≥2 points) during pregnancy (online supplemental figure 12). For example, we found a large discrepancy at 34 weeks' gestation when the Scottish and Irish MEWS triggered escalation of care at least twice as often as the new national MEWS (new national MEWS 18%, Scottish MEWS 36%, and Irish MEWS 43%).

#### Early antenatal adjustments

The early antenatal specific threshold values (online supplemental table 1 and online supplement file 1) were similar to those of the new national MEWS (online supplemental figure 13). For the early antenatal MEWS, 53% of observations scored 0 points, 23% scored 1 point, 17% scored 2 points, and 24% scored ≥2 points (online supplemental figure 14). The new national MEWS had similar scores: 52% scored 0 points, 28% scored 1 point, 12% scored 2 points, and 20% scored ≥2 points. Because the early antenatal threshold values were sufficiently similar to the new national MEWS, and the relative proportions in each scoring category were not affected, we concluded that the new national MEWS was appropriate to use in the early antenatal period without adjustment.

#### Early postnatal adjustments

The early postnatal specific threshold values (online supplemental table 2 and online supplement file 1) were similar to those of the new national MEWS, with the exception of heart rate and temperature (online supplemental figure 13). The heart rate threshold values in the early postnatal period were shifted about 10 beats/min lower, whereas the temperature threshold values were shifted about 0.25 °C higher. The early postnatal specific threshold values scored 61% of observations as 0 points, 24% as 1 point, and 14% as ≥2 points (online supplemental figure 15). The new national MEWS scored 40% of observations as 0 points, 30% as 1 point, and 30% as ≥2 points.

The proportion of observations with a score of ≥2 points with the new national MEWS (30%) in the early postnatal period was higher than in the rest of pregnancy (24% in the early antenatal period and 18% in the later antenatal period). Most (98%) heart rate alerts according to the new national MEWS were for low heart rate. Incorporating the postnatal heart rate threshold values into the new national MEWS gave overall alerting rates similar to other stages of pregnancy, with 56% scored 0 points, 26% scored 1 points, 13% scored 2 points, and 18% scored ≥2 points. Changing the temperature threshold values made minimal difference to alerting rates (online supplemental figure 15). Therefore, for simplicity, we chose to keep the original new national MEWS temperature threshold values. Table 3 shows a modified version of the new national MEWS for postnatal use.

**Table 3:** New national MEWS with postnatal modification

Vital signs	Score				
	2 points	1 point	0 points	1 point	2 points
Respiratory rate (breaths/min)	≤6	7-8	9-21	22-24	≥25
Oxygen saturation (%)	≤92	93-94	≥95	—	—
Temperature (°C)	≤34.8	34.9-35.5	35.6-37.2	37.3-37.5	≥37.6
Pulse rate (beats/min)	≤50	51-57	58-98	99-107	≥108
Systolic blood pressure (mm Hg)	≤93	94-100	101-135	136-144	≥145
Diastolic blood pressure (mm Hg)	≤56	57-61	62-88	89-96	≥97

Scoring threshold values recommended for use in the postnatal period. Scores are the same as the antenatal threshold values, except for pulse rate. Interpretation of scores is the same as described in table 2.

MEWS, maternity early warning score.

### Delphi consensus exercise

#### Delphi round 1

We invited 67 participants to take part in the consensus exercise. Online supplemental table 4 summarises the clinical roles of the candidates. We received responses from 56 of the candidates (84%). Responses were received over a three week period in February 2021.

#### Delphi round 2

Of the 56 candidates who responded to round 1 and were therefore invited to complete round 2, we received responses from 43 (77%) of the candidates. Online supplemental figure 16 shows the suggested clinical responses reached by consensus for each MEWS. For the primary response, the consensus of opinion was that all MEWS, other than score 0 points with no additional concern, prompted contact with the midwife. Contact with a clinician was suggested at a MEWS of 2 points, with a graded escalation of clinical seniority as MEWS increased. Secondary responses mirrored the primary responses, escalated by one tier of clinical seniority.

**Figure 3:**
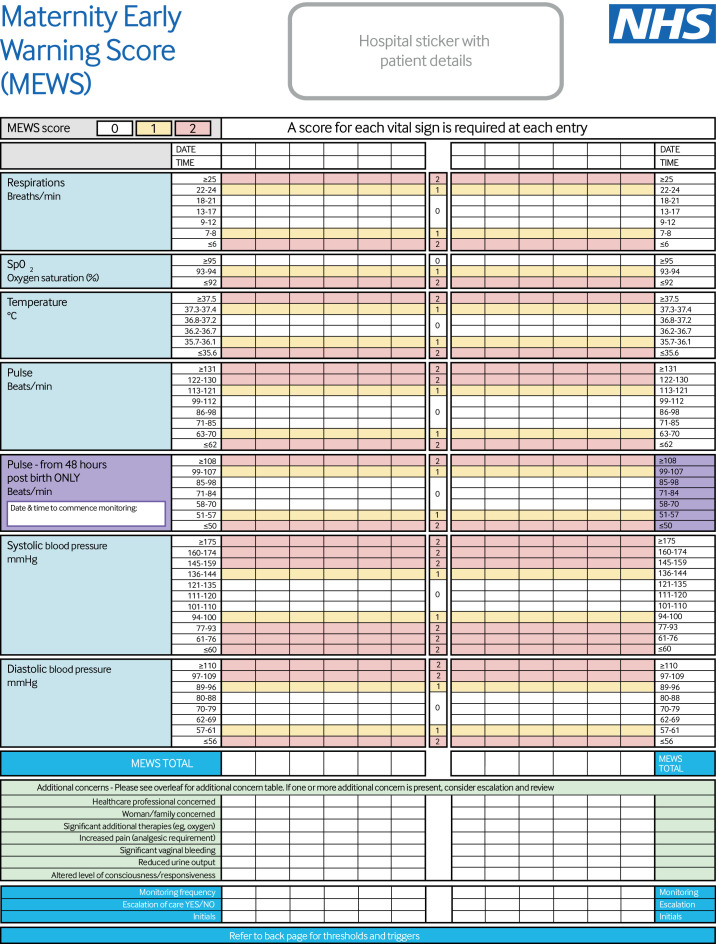
Final formatted version of new national maternity early warning score (MEWS): front page

**Figure 4:**
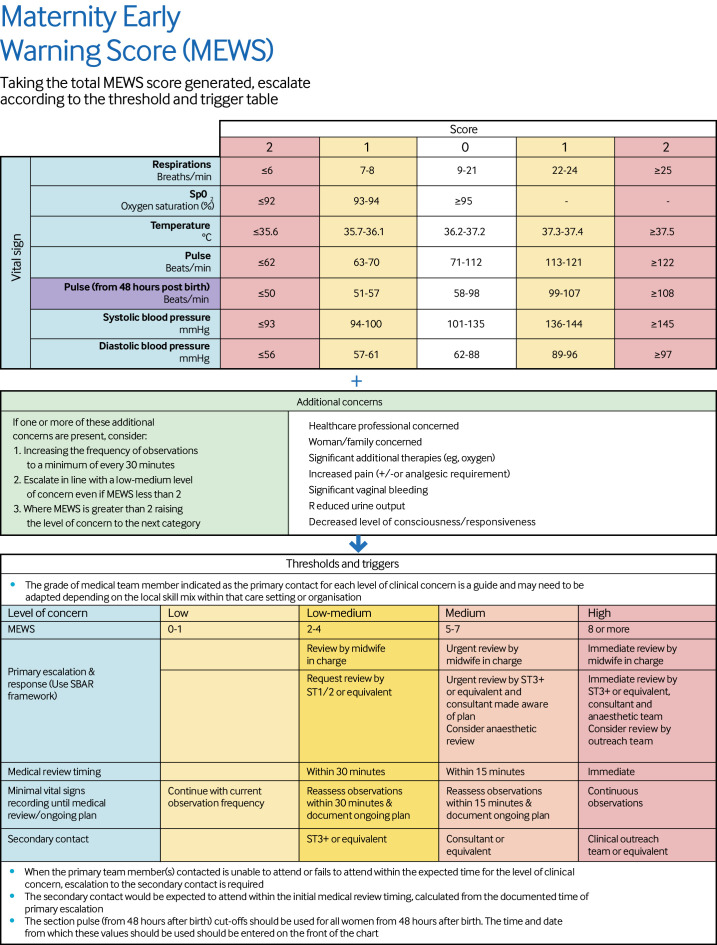
Final formatted version of new national maternity early warning score (MEWS): back page, showing threshold values and triggers. Specialty trainee (ST) 1-2 and 3+ had ≤2 and ≥3 years of obstetric training, respectively. SBAR=situation, background, assessment, and recommendation

Online supplemental figure 17 shows the suggested changes to the frequency of observations reached by consensus for each score. A MEWS of 0 points resulted in no change, whereas for a MEWS of 0 points with concern, escalation to hourly observations was suggested. The consensus of opinion was that the frequency of observations should then increase incrementally at MEWS of 1 point (repeat in 30 minutes), 5 points (repeat in 15 minutes), and 8 points (move to continuous monitoring). The suggested responses produced from the Delphi consensus exercise were reviewed and ratified by the same core development group from earlier in the process. Figures 3 and 4 show the final MEWS.

## Discussion

### Principal findings

Our new national MEWS was developed based on data from a prospective observational study. The MEWS scoring threshold values were calculated based on the modelled distributions of each vital sign during the antenatal and immediate postnatal periods. We ensured a clinically appropriate and acceptable triggering rate by using centiles to determine the threshold values. We determined appropriate escalation responses to MEWS values with a Delphi process with multidisciplinary expert stakeholders.

The new national MEWS had a narrower range of normal (score 0 points) for systolic and diastolic blood pressure than the Scottish and Irish MEWS. Low diastolic blood pressure was considerably more likely to score with the new national MEWS. For heart rate and temperature, the normal regions of the new national MEWS were similar in size to the Scottish and Irish MEWS but were shifted. For heart rate, the normal range included higher values, whereas for temperature, lower values were included. The normal ranges for respiratory rate and SpO_2_ in the new national MEWS were larger in size than the Scottish and Irish MEWS. For SpO_2_, the normal range extended to 94%, the same as in the Scottish MEWS (95% for Irish MEWS), but the new national MEWS also included a region that scored 1 point, consistent with other vital signs, unlike the other two MEWS.

A key characteristic of the new national MEWS is that each vital sign has a broadly similar probability of scoring (ie, each vital sign is weighted about equally). This balance was achieved by choosing the threshold values based on the observed distribution of each vital sign in the 4P cohort. Because MEWS are currently more a measure of physiological normality than a true prognostic model, this weighting seems appropriate. SpO_2_ differed from the other observations, alerting escalation of care in 6% of observations from healthy women (rather than about 10% for other observations). This finding is because our early warning score was derived from observations at about 34 weeks' gestation when SpO_2_ values are at their lowest. Because only low oxygen saturation values, rather than both low and high saturation values, alerted escalation of care as with other vital signs, this might be appropriate, but could change in the future if larger datasets become available with well documented outcomes that enabled a true prognostic model to be developed.

Because of the varied points scoring across the vital signs for the three MEWS, the distributions of the total scores differed. With the new national MEWS, 18% of all observations in a healthy population triggered escalation of care, compared with 38% with the Irish tool and 32% with the Scottish MEWS. The triggering rate of the new national MEWS was more in line with the proportion of observations that meet the national early warning score threshold of 5 points in general adult hospital inpatients, estimated to be about 20%.^24^ Over the course of their pregnancy, more than half of the women in our dataset would have had escalation of care with either the Scottish or Irish MEWS, assuming an escalation threshold of 2 points, rather than about 30% with the new national MEWS. These high rates of escalation with the Scottish and Irish MEWS might lead to fatigue among midwives, and therefore a breakdown of the clinical response pathway.^25 26^

In current practice, MEWS are primarily used in the peripartum period and hence our threshold values for the new national MEWS were based on the distribution of vital signs at 34 weeks' gestation. Escalation rates in healthy women were constant over pregnancy with the new national MEWS but became increasingly frequent as pregnancy progressed with the other tools. We found that during the third trimester, the difference between the new national MEWS and the Scottish and Irish MEWS was particularly large. The mean total score with the Irish MEWS was about double that of the new national MEWS for the whole of the third trimester. We have shown high escalation rates in healthy individuals when the Scottish and Irish scores were used based on data averaged across the whole of pregnancy. At around 34 weeks' gestation, the rate of triggering escalation of care for the Scottish and Irish MEWS in healthy women was at least double that of the new national MEWS (new national MEWS 18%, Scottish MEWS 36%, and Irish 43% MEWS, online supplemental figure 12). Previous studies based on the 4P data have shown that moderate changes exist in the normal ranges for some vital signs during pregnancy.^8 17^ For example, normal heart rate values rise during pregnancy, whereas oxygen saturation and temperature fall. Changes in blood pressure are also seen, particularly in the later stages of pregnancy. The additive effect of these changes in normal vital signs cause the average Scottish and Irish total scores to rise during pregnancy. That the average new national MEWS score remained constant throughout pregnancy was probably because different vital signs are more likely to score during different stages of pregnancy, with changes broadly balancing each other out. Future work might include a gestational age specific MEWS.

### Strengths and limitations of this study

We used a Delphi process to establish consensus informed escalation procedures. We kept the independence of the panellist throughout, which ensured each participant's opinion carried equal weight, and opinions could not be swayed by the individual opinions of another; opinions were informed only by the group consensus after round 1. Panellists were selected purposively by a group external to the authors to include individuals with relevant but varied experience of maternity care. This method produced a MEWS with advised escalation responses systematically derived from consensus of a large multidisciplinary expert panel ensuring high construct validity.^20 27^

After this development process, the tool underwent iterative rounds of development and testing, including a round of prototype testing with 54 healthcare professionals, followed by a round of testing on a set of cold cases. This process involved 16 organisations across England. In subsequent rounds of testing, the tool was used alongside current tools in a range of clinical environments in 13 organisations.

Some limitations should be considered when evaluating the new national MEWS. The threshold levels were based on normal ranges at 34 weeks' gestation, representing the midpoint in the third trimester. Values for vital signs are relatively constant throughout the third trimester, however, so varying the exact point would not have a large effect. The tool was not validated against adverse outcomes, which would require a large dataset, so we do not know how well the score could differentiate between women with good and poor outcomes. We recommend that a true validation study based on a large dataset should be carried out as the next step. Women in the study were recruited from three UK centres and most (85%) were white women. Therefore, the generalisability of the score could be questioned, further emphasising the need for external validation. Also, the performance of the score was assessed based on the same data used to develop the score, which might result in an optimistic assessment. As well as external validation of the new MEWS score, future studies might also develop a more sophisticated model which: might include more variables, such as personal characteristics and laboratory tests; give a probability estimate of a woman's risk of a poor outcome; and retain the continuous nature of predictor variables. Although we propose adjustment to the heart rate values used to alert to deterioration in the early postpartum period, the possibility that further improvements could be obtained by considering variations in other vital signs throughout pregnancy needs to be explored. Our new national MEWS already represents a step up in the level of complexity, and we were aware that adding further complexity would require a fully electronic based scoring system. Despite some limitations, we believe our approach to developing an evidence based alerting system and response pathway moves this field forward.

### Study implications for research and practice

Our MEWS and escalation systems could be adapted to international settings with a few modifications. Recent systematic reviews have reported that the vital signs used in our MEWS are routinely recorded during pregnancy and in the peripartum period in different international healthcare systems.^2 5^ In fact, most studies of the effect of implementation of MEWS on outcomes were conducted in low resource settings.^5^ Specialty training programmes are broadly similar across Europe, Australia, and North America, and therefore appropriately skilled clinicians can be easily translated from their UK equivalents for each escalation threshold.^28^

### Conclusions

This MEWS was developed based on normal ranges derived from patient data. Its use should considerably reduce alerts in healthy women compared with other MEWS tools. Unlike other MEWS systems, appropriate escalation responses were derived with a systematic, expert informed process. The complete MEWS tool was ratified by an independent expert panel. We could not adequately assess the performance of our new national MEWS, however, because of the small size of our dataset. We recommend external validation of the new national MEWS.

10.1136/bmjmed-2023-000748.supp2 Supplementary data

## Data Availability

Data are available upon reasonable request. The data used in this manuscript are from the Pregnancy Physiology Pattern Prediction (4P) study.
