# Supplementary material for: Development of a national maternity early warning score: centile based score development and Delphi informed escalation pathways
Source: BMJ Med. 2024 May 15;3(1):e000748. doi: 10.1136/bmjmed-2023-000748 (PMC11097818; doi:10.1136/bmjmed-2023-000748)
Supplement: Supplementary data [file bmjmed-2023-000748supp001.pdf]

Supplementary material

1 Modifications in early antenatal period (<18 weeks gestation)

The early antenatal-specific thresholds (Supplementary Table 1) were similar to those of the New MEWS (Supplementary Figure 13). Only for SBP and DBP were any notable differences observed, with the early antenatal ranges slightly lower than the New MEWS (a shift of approximately 5mmHg for both SBP and DBP). The National Early Warning Score (NEWS) thresholds were markedly different from both, in particular with respect to SBP, HR, and SpO_2. The early antenatal MEWS scores 53% of observations zero, 23% one, 17% two, and 24% two or more. The New MEWS scores 52% zero, 28% one, 12% two, and 20% two or more. NEWS scores 25% zero, 39% one, 19% two, and 36% two or more (Supplementary Figure 14).

Since the early antenatal thresholds are sufficiently like the New MEWS and there is little impact on the relative proportions in each scoring category, we concluded that the New MEWS was appropriate to use in the early antenatal period without adjustment.

Supplementary Table 1: The scoring thresholds based on the early antenatal period (16 weeks gestation)

Vital sign	Two	One	Zero	One	Two
Systolic Blood Pressure	≤89	90-96	97-133	134-142	≥143
Diastolic Blood Pressure	≤52	53-56	57-83	84-90	≥91
Pulse	≤59	60-66	67-103	104-113	≥114
Respiratory Rate	≤6	7-8	9-21	22-24	≥25
SpO2	≤93	94-95	≥96		
Temperature	≤35.0	35.1-35.6	35.7-37.3	37.4-37.6	≥37.7

2 Modifications in early postnatal period (first 16 days after delivery)

The early postnatal-specific thresholds (Supplementary Table 2) were similar to those of the New MEWS, with the exception of heart rate and temperature (Supplementary Figure 13). The HR thresholds are shifted approximately 10 BPM lower, whereas the temperature thresholds are shifted approximately 0.25 degrees Celsius higher. The NEWS thresholds differ considerably to those of the New MEWS. The early postnatal-specific thresholds score 62% of observations as zero, 24% one, 10% two, and 14% two or more (Supplementary Figure 15). The New MEWS scores 40% zero, 30% one, 20% two, and 30% two or more. NEWS scores 42% zero, 36% one, 15% two, and 22% two or more.

The proportion of observations with a score of two or more with the New MEWS (30%) was considered too high, since we were aiming for approximately 20%. Whilst the rate for NEWS was better (22%), the rate scoring zero was low (42%). The aim, for the purpose of consistency, was to stay as close to the New MEWS as possible, therefore we investigated a few minimal changes. We concluded that using the New MEWS, but with the postnatal HR thresholds, gave a much better scoring rate, with 56% zero, 26% one, 13% two, and 18% two or more. Changing the temperature thresholds made minimal difference, and therefore we chose to keep the original New MEWS thresholds for temperature.

Supplementary Table 2: The scoring thresholds based on the post-natal period (5 days after delivery)

Vital sign	Two	One	Zero	One	Two
Systolic Blood Pressure	≤96	97-103	104-138	139-146	≥147
Diastolic Blood Pressure	≤59	60-64	65-91	92-97	≥98
Postnatal Pulse	≤50	51-57	58-98	99-107	≥108
Respiratory Rate	≤6	7-8	9-21	22-24	≥25
SpO2	≤92	93-94	≥95		
Temperature	≤35.3	35.4-35.8	35.9-37.6	37.7-37.9	≥38.0

3 Supplementary Tables

Supplementary Table 3: Pregnancy complications and birth outcomes according to the period of pregnancy for the data used to evaluate model performance

Complications and Outcomes	Early antenatal < 18 weeks gestation [n=841]	Antenatal 18-40 weeks [n=998]	Postnatal 0-16 days post-delivery [n=889]
Gestational diabetes	32 (3.8)	38 (3.8)	31 (3.5)
Gestational hypertension	32 (3.8)	46 (4.6)	45 (5.1)
Preeclampsia	17 (2.0)	18 (1.8)	15 (1.7)
Severe preeclampsia, HELLP, eclampsia	4 (0.5)	5 (0.5)	3 (0.3)
Termination of pregnancy at less than 12 wk	0 (0)	0 (0)	0 (0)
Termination of pregnancy at more than 12 wk	3 (0.4)	0 (0)	0 (0)
Miscarriage at less than 12 wk	1 (0.1)	0 (0)	0 (0)
Late miscarriage 12-24 wk	10 (1.2)	3 (0.3)	0 (0)
Intrauterine death or stillbirth at more than 24 wk	3 (0.4)	3 (0.3)	0 (0)
Preterm delivery at less than 37 0/7 wk	58 (6.9)	63 (6.3)	46 (5.2)

Spontaneous vaginal birth	512 (60.9)	613 (61.4)	560 (63.0)
Assisted vaginal birth	134 (15.9)	156 (15.6)	144 (16.2)
Cesarean birth	166 (19.7)	204 (20.4)	178 (20.0)
Birth weight at 37 0/7 wk or more (g)	3420 (474)	3430 (483)	3440 (489)
Term low birth weight (less than 2,500 g; 37 0/7 wk or more	13 (1.5)	17 (1.7)	16 (1.8)
HELLP, hemolysis, elevated liver enzymes, low platelet count<br>Data are n() or mean ± SD			

4 Supplementary Figures

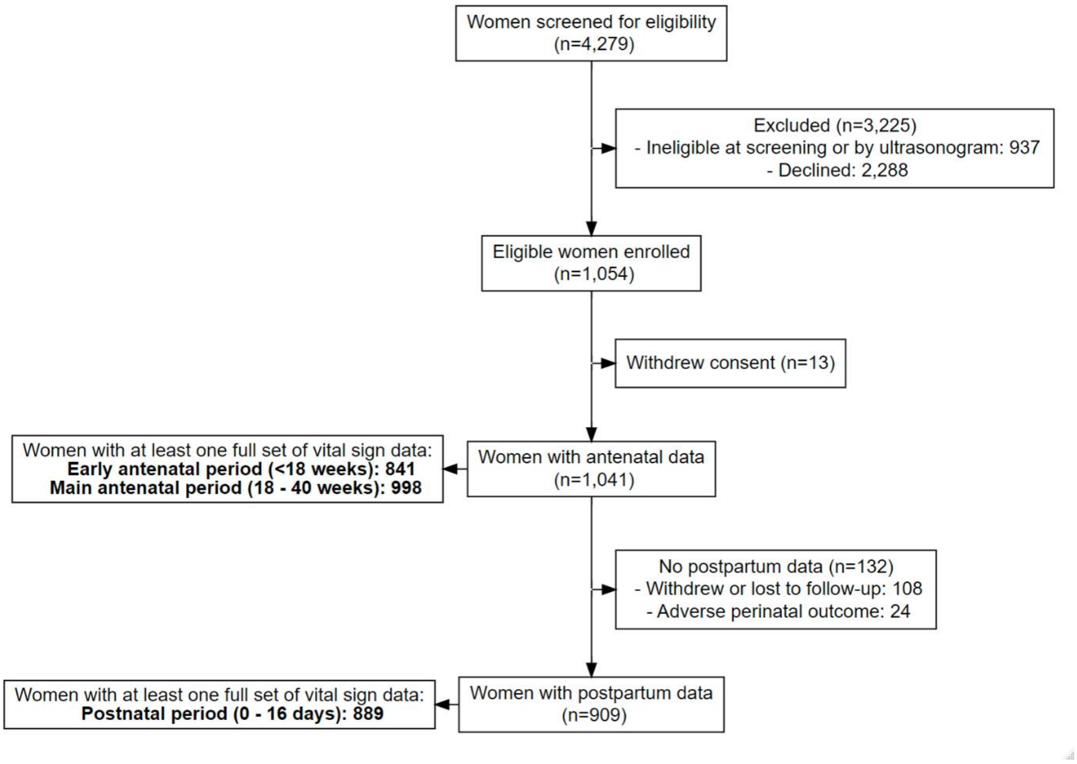

Supplementary Figure 1: Flow chart of participants through the study

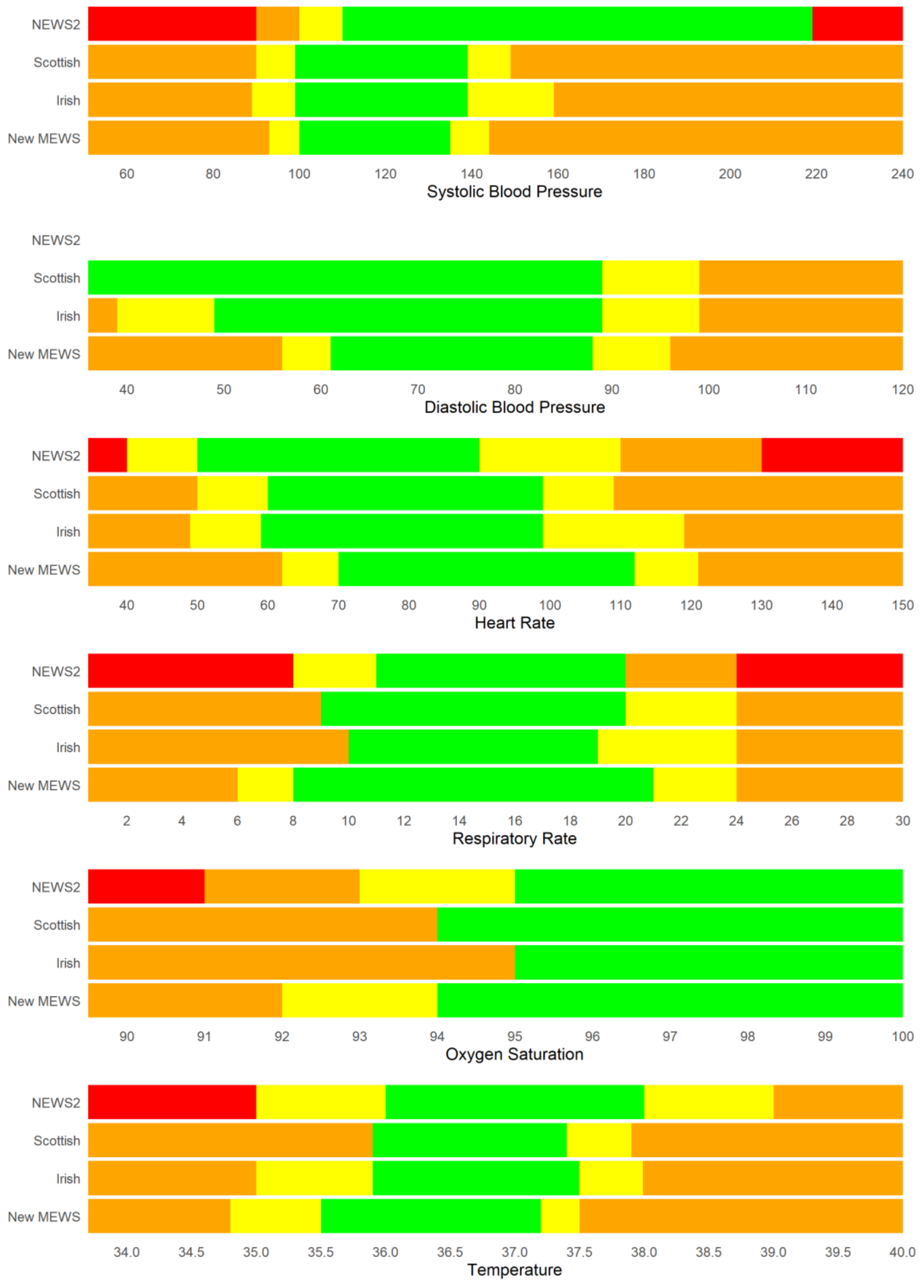

NB: NEWS2 does not include diastolic blood pressure

Supplementary Figure 2: A visual depiction of the scoring thresholds of the New MEWS, Scottish MEWS, Irish MEWS, and NEWS2, for each vital sign. Green areas score zero points, yellow areas score one point, orange areas score two points, and red areas score three points.

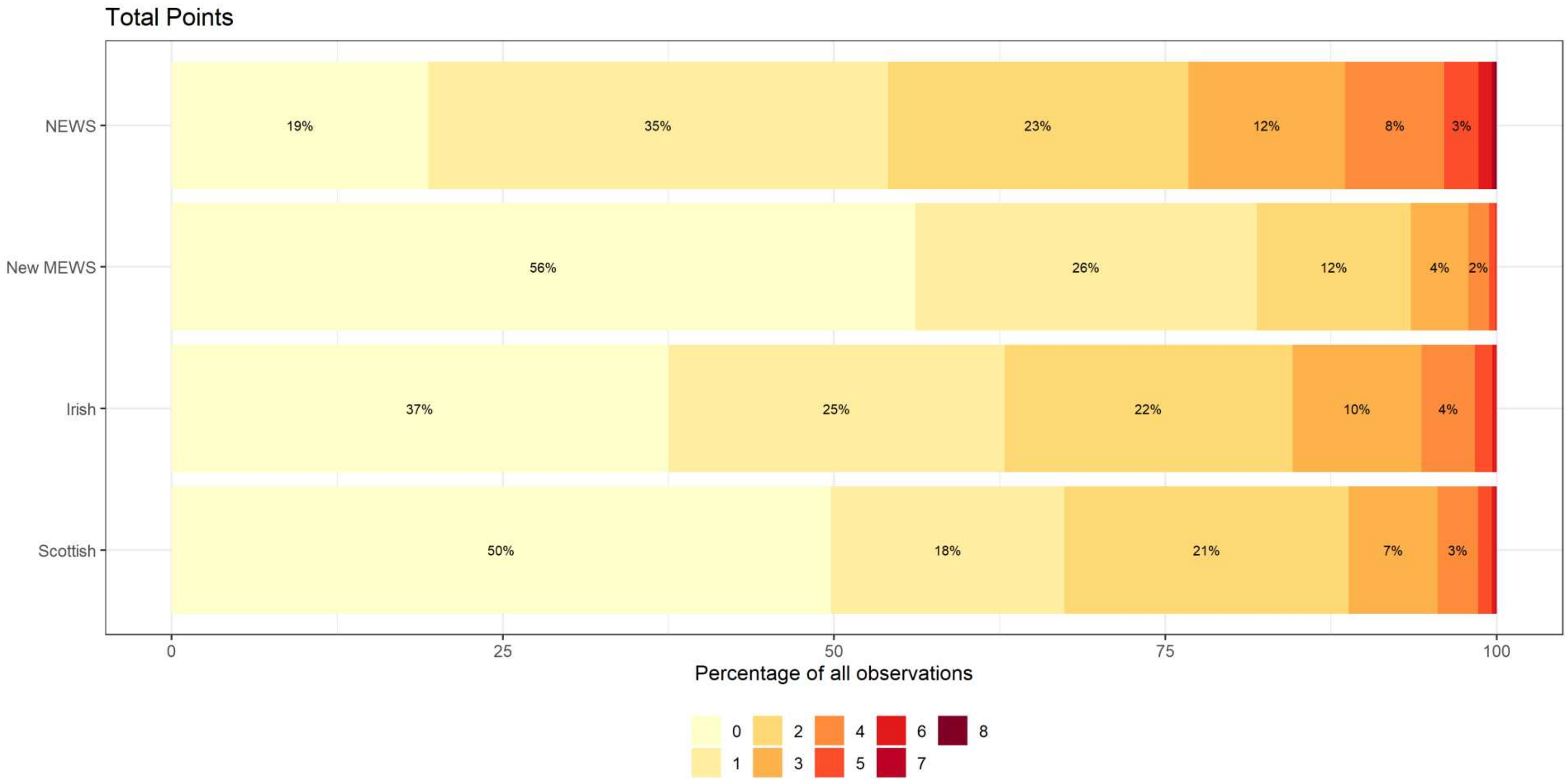

Supplementary Figure 3: The distribution of total Early Warning Scores for the New MEWS, Scottish MEWS, Irish MEWS, and NEWS2, in all observation sets.

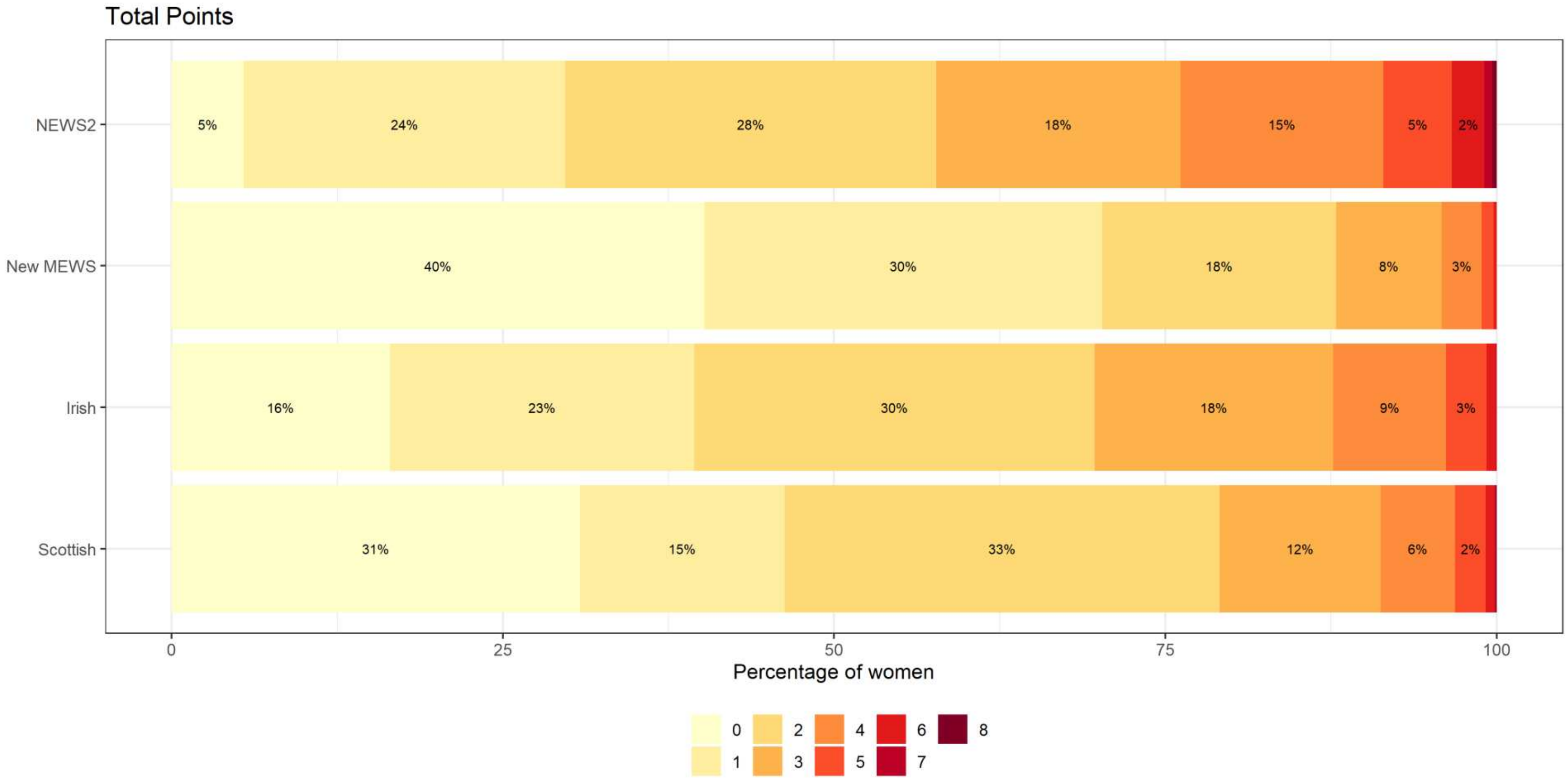

Supplementary Figure 4: The distribution of a woman's highest total Early Warning Score for the New MEWS, Scottish MEWS, Irish MEWS, and NEWS2

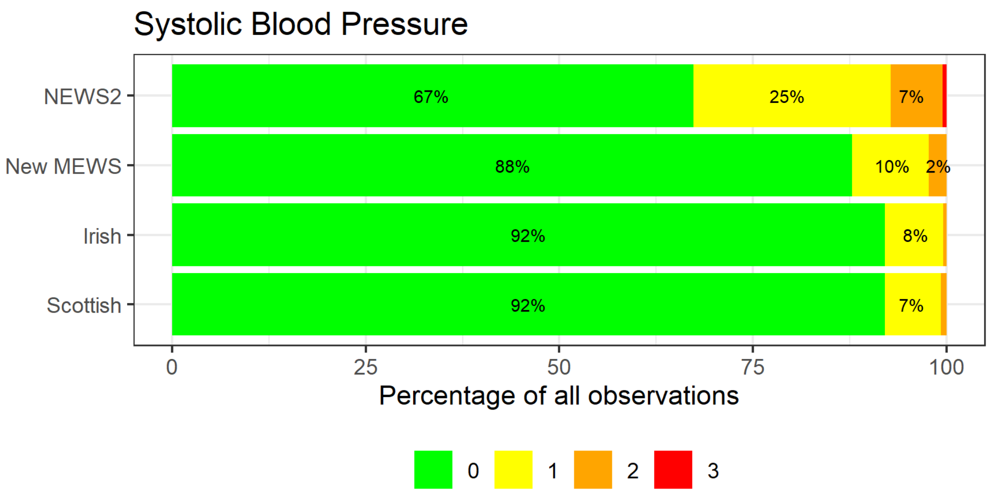

Supplementary Figure 5: The distribution of systolic blood pressure EWS points for the new MEWS, Scottish MEWS, Irish MEWS, and NEWS2, in all observation sets.

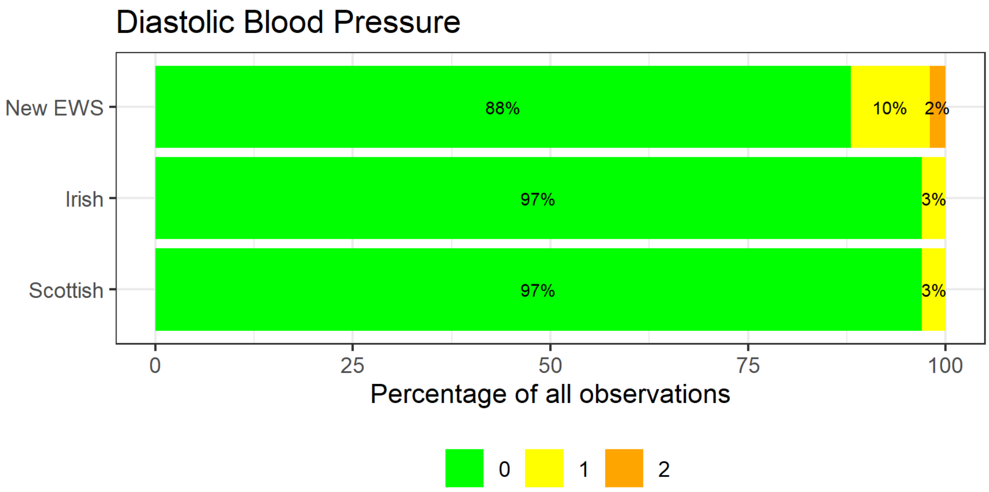

NB: NEWS2 does not include diastolic blood pressure

Supplementary Figure 6: The distribution of diastolic blood pressure EWS points for the new MEWS, Scottish MEWS, Irish MEWS, and NEWS2, in all observation sets.

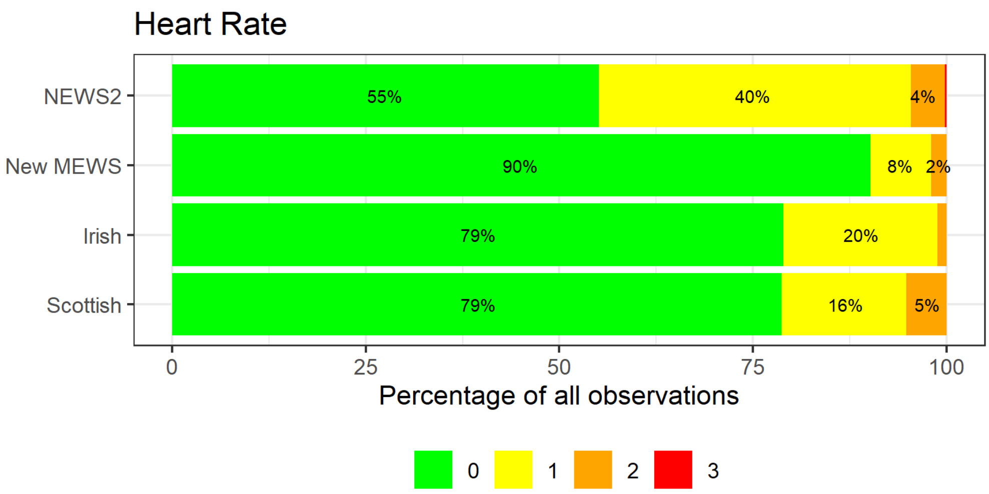

Supplementary Figure 7: The distribution of heart rate EWS points for the new MEWS, Scottish MEWS, Irish MEWS, and NEWS2, in all observation sets.

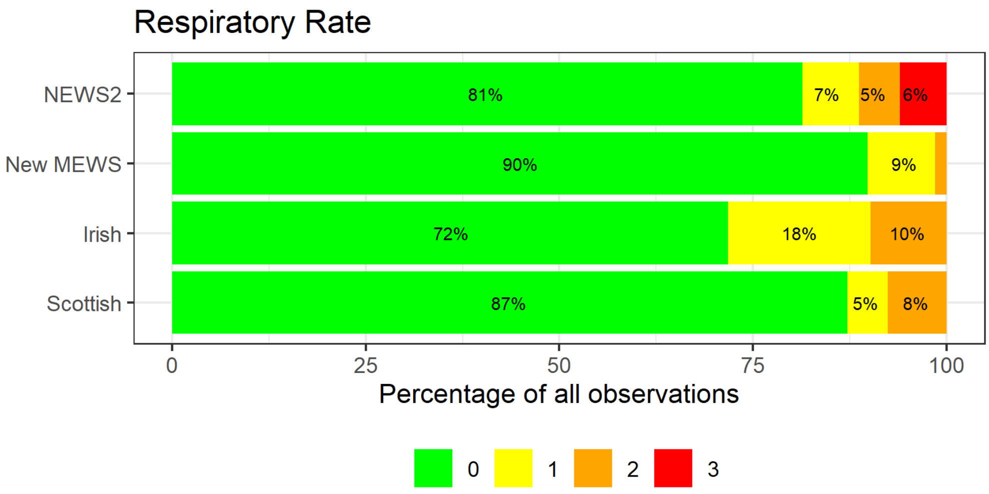

Supplementary Figure 8: The distribution of respiratory rate EWS points for the new MEWS, Scottish MEWS, Irish MEWS, and NEWS2, in all observation sets.

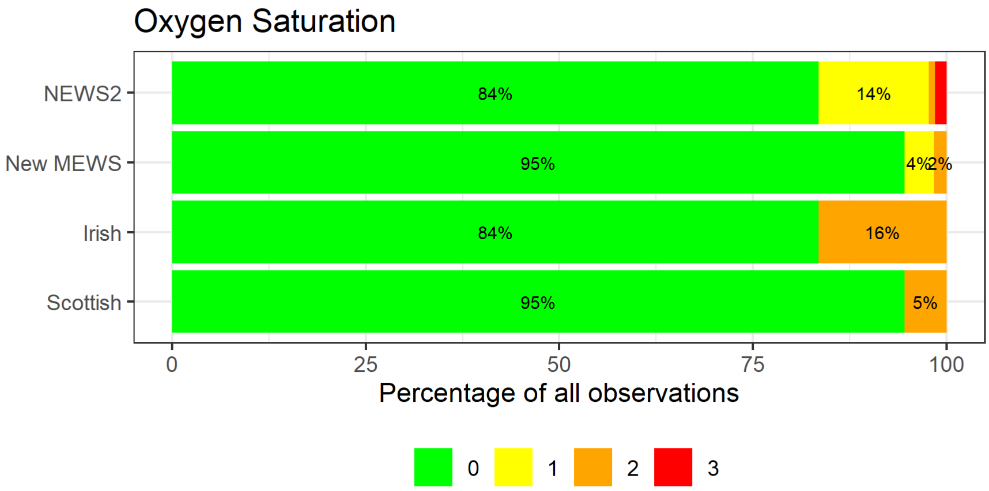

Supplementary Figure 9: The distribution of oxygen saturation EWS points for the new MEWS, Scottish MEWS, Irish MEWS, and NEWS2, in all observation sets.

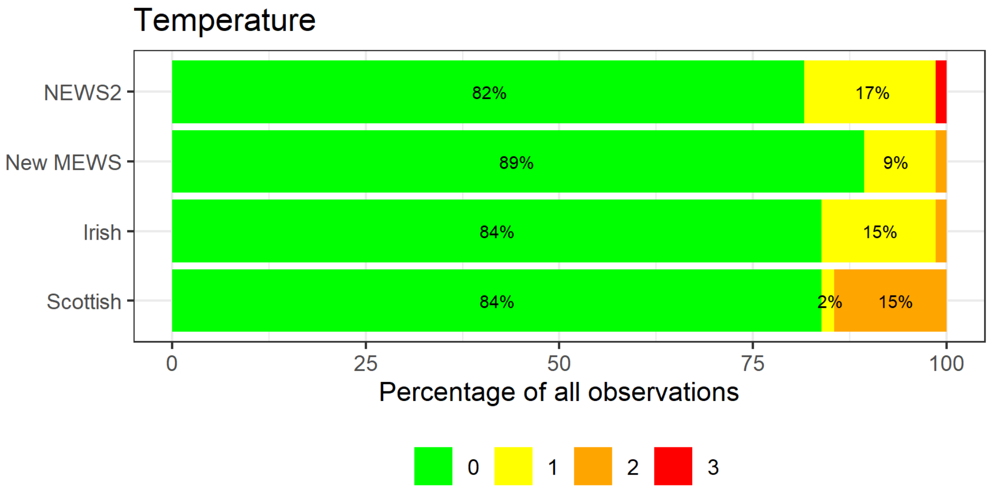

Supplementary Figure 10: The distribution of temperature EWS points for the new MEWS, Scottish MEWS, Irish MEWS, and NEWS2, in all observation sets.

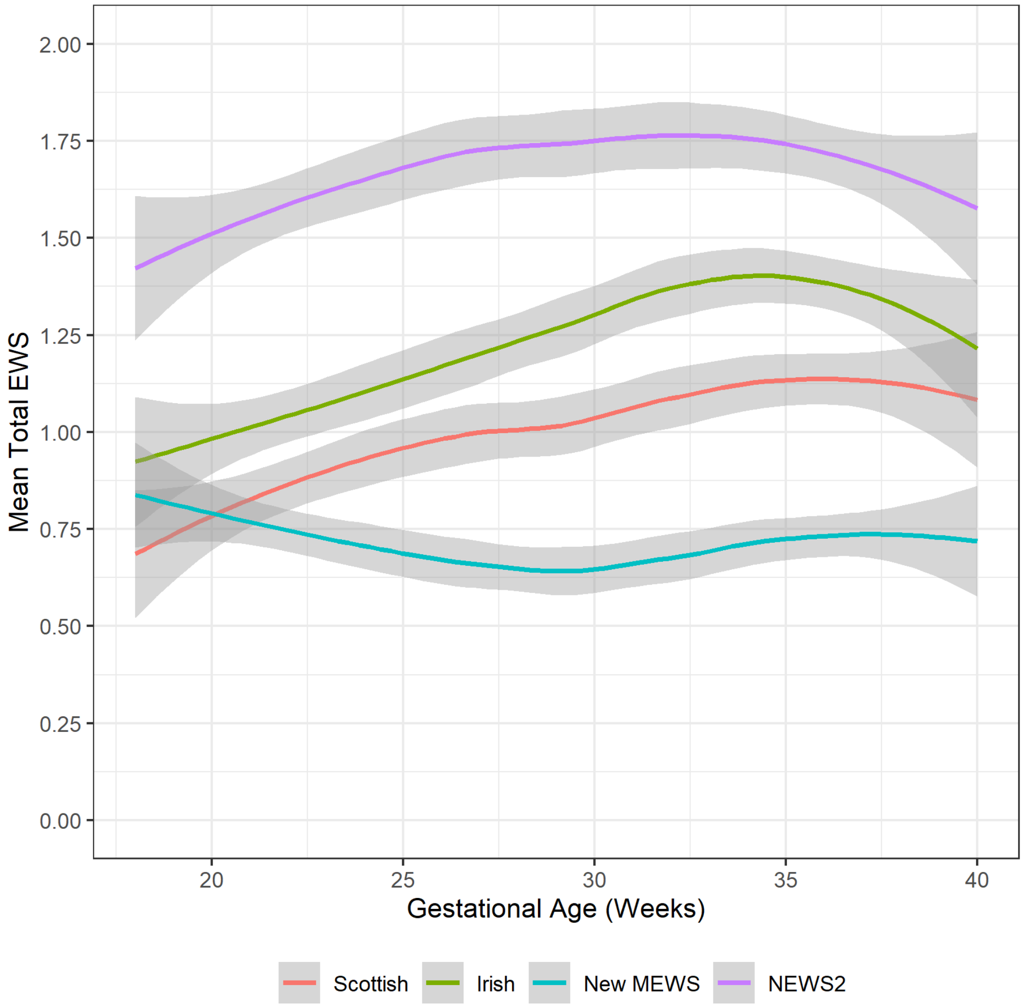

Supplementary Figure 11: The mean total EWS across gestational ages for the new MEWS, Scottish MEWS, Irish MEWS, and NEWS2 separately. Curves created using LOESS (locally weighted smoothing) smoother.

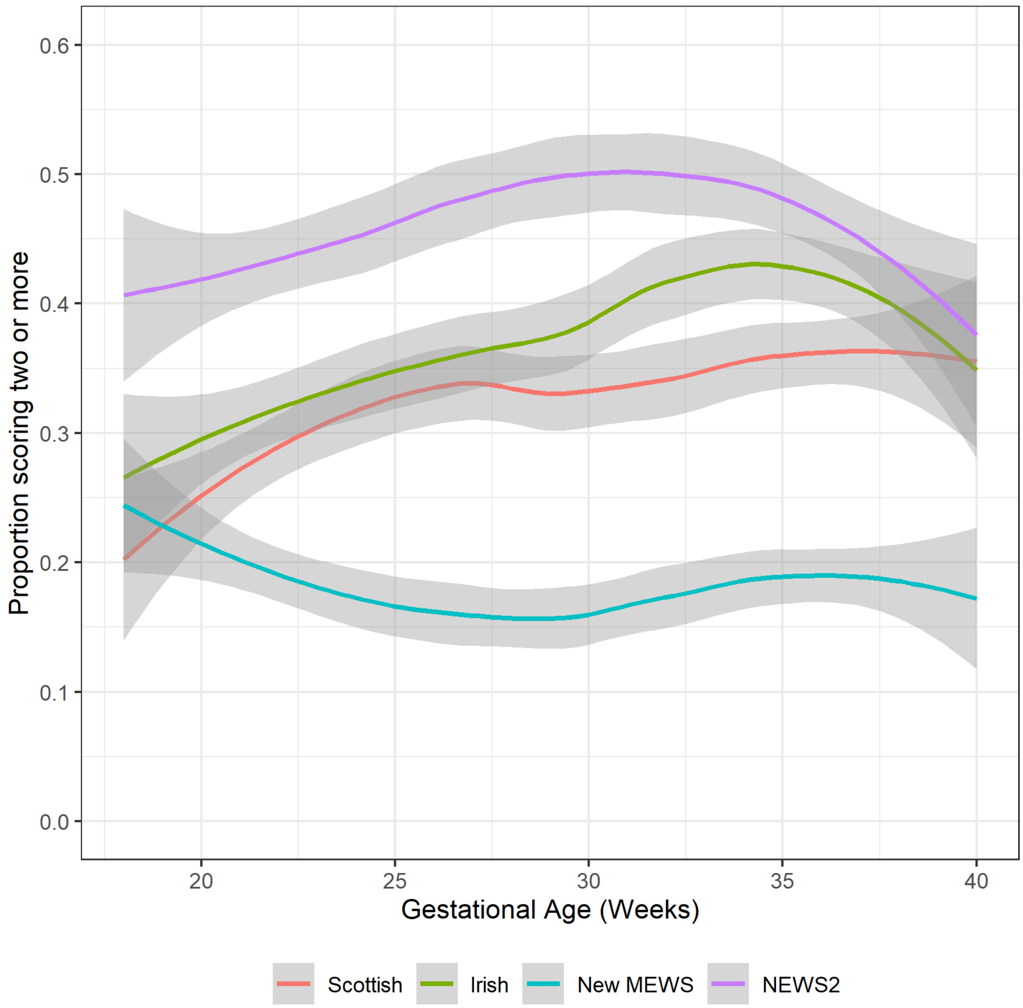

Supplementary Figure 12: The proportion of total EWSs that trigger (i.e., score two or more points) across gestational ages for the new MEWS, Scottish MEWS, Irish MEWS, and NEWS2 separately. Curves created using LOESS (locally weighted smoothing) smoother.

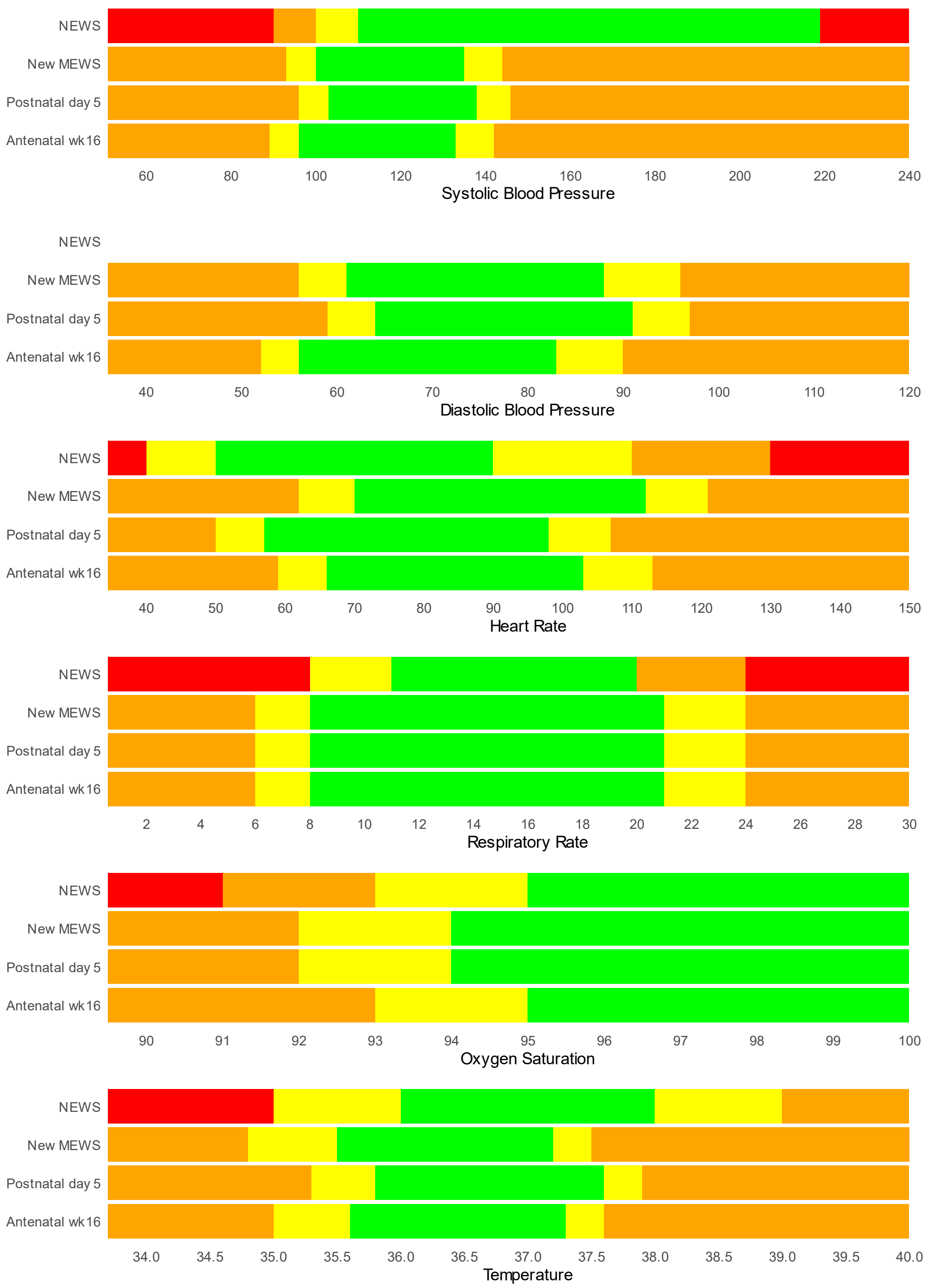

NB: NEWS2 does not include diastolic blood pressure

Supplementary Figure 13: A visual depiction of the scoring thresholds of the new mEWS, the early antenatal EWS, the early postnatal EWS and NEWS, for each vital sign. Green areas score zero points, yellow areas score one point, orange areas score two points, and red areas score three points.

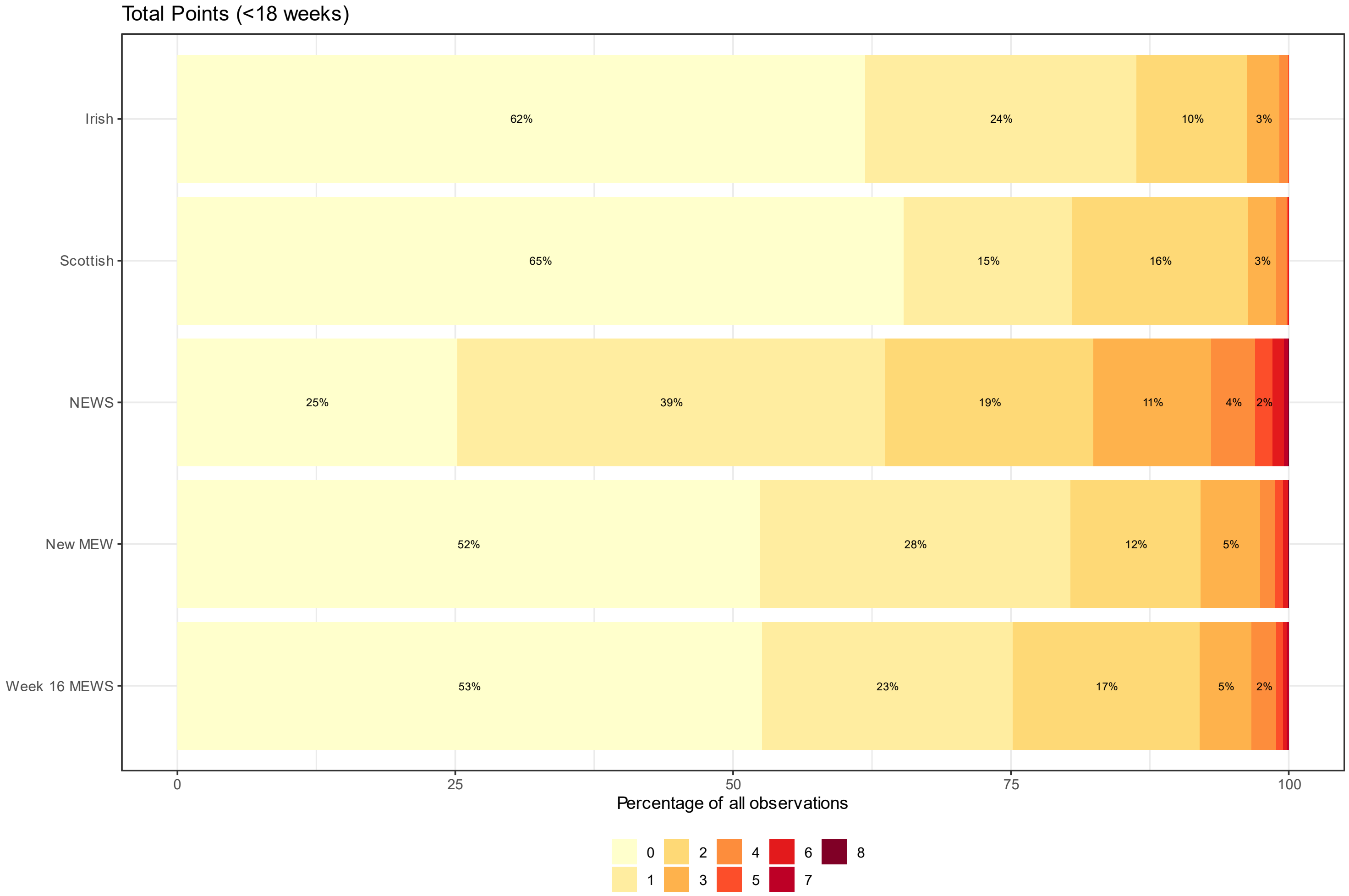

Supplementary Figure 14: The distribution of total Early Warning Scores for the New MEWS, Scottish MEWS, Irish MEWS, the early antenatal MEWS, and NEWS2, in all observation sets from the early antenatal period.

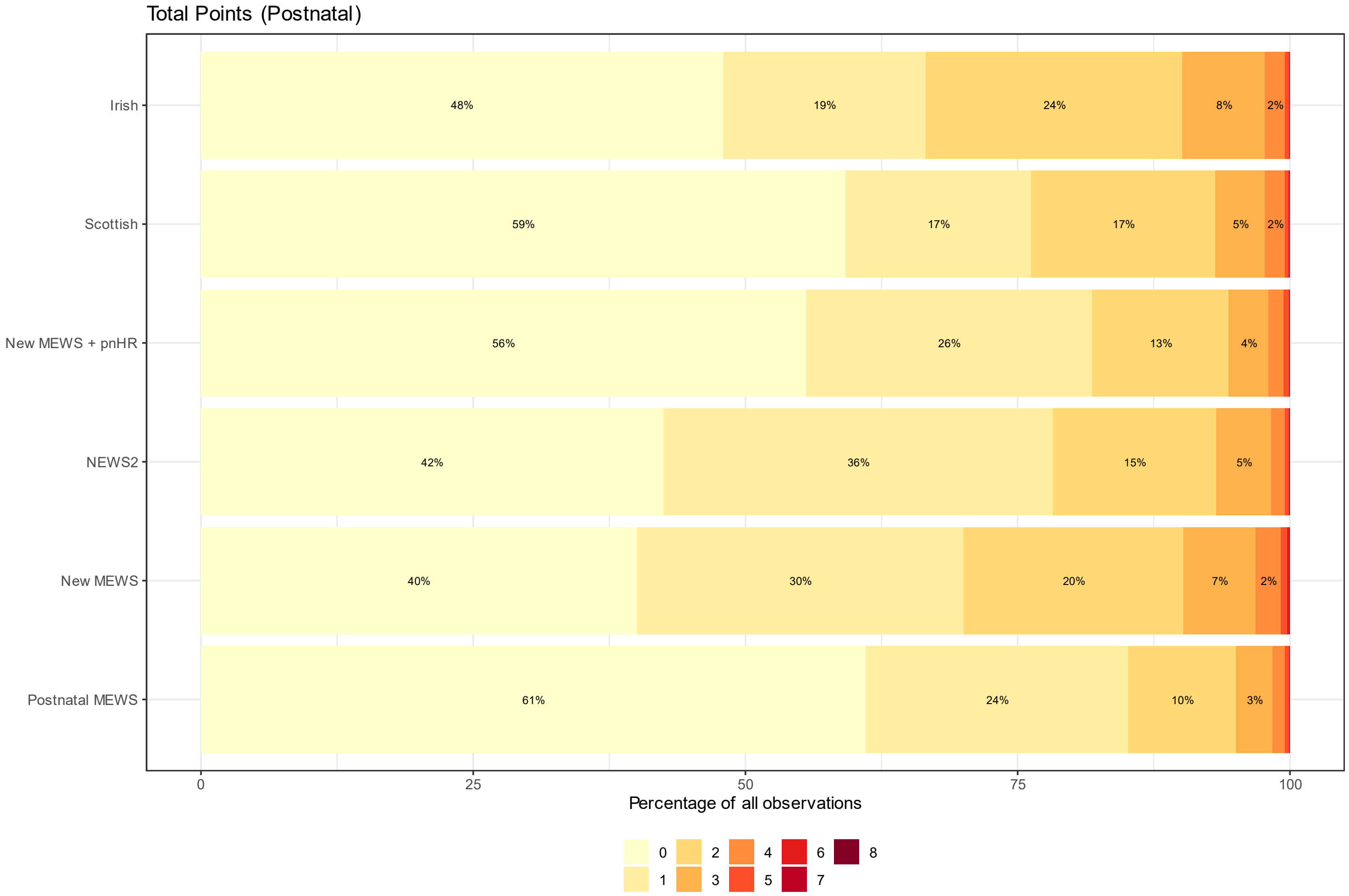

Supplementary Figure 15: The distribution of total Early Warning Scores for the New MEWS, Scottish MEWS, Irish MEWS, the early postnatal MEWS, NEWS2, and the New MEWS + postnatal HR, in all observation sets from the postnatal period.

5 Additional Delphi Information

Supplementary Table 4 - Roles of Delphi candidates

Job Role	Count
Midwife	38
Consultant Obstetrician	15
Clinician Researcher	3
Specialist Registrar - Obstetrics	3
Consultant Midwife	3
Specialist Registrar - Maternal Medicine	2
Consultant Obstetric Physician	1
Head of Midwifery and Maternity Research	1
Senior NHS Improvement Manager	1

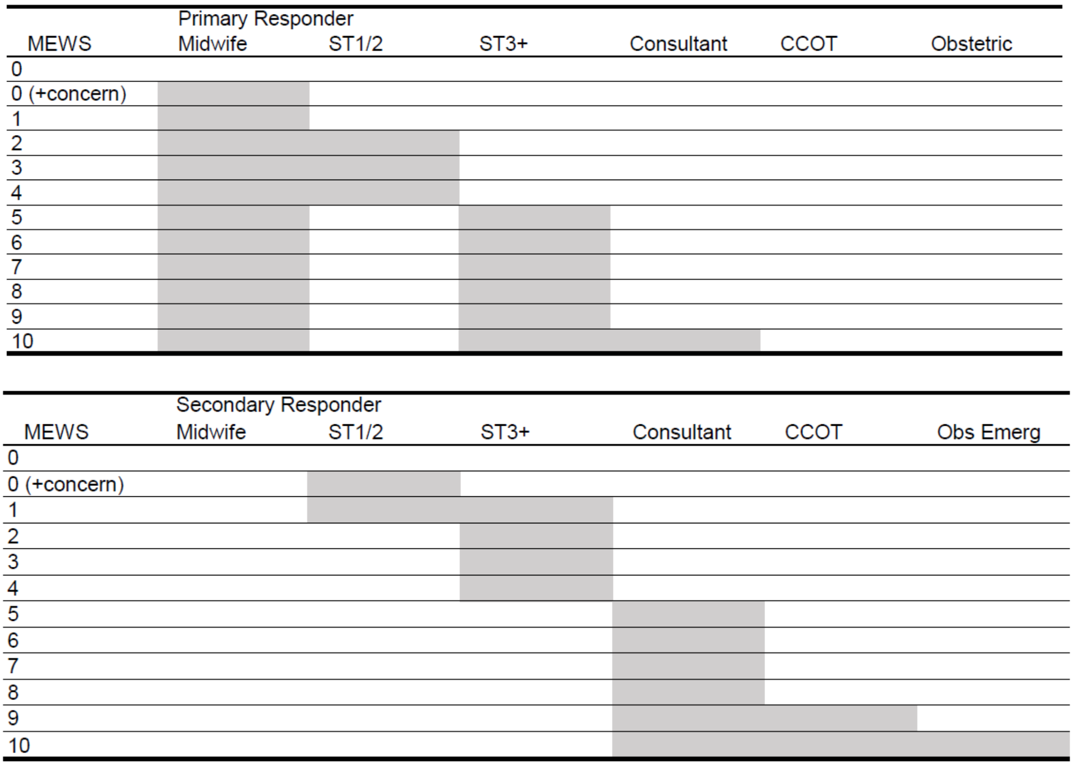

Supplementary Figure 16: Consensus-suggested primary contacts (top panel) for a given MEWS. Secondary contacts (bottom panel) were sought in the case where the primary contact did not attend within the appropriate time

	Frequency of observations				
mEWS	No Change	Repeat in 1 h	Repeat in 30 mins	Repeat in 15 mins	Continuous
0					
0 + concern					
1					
2					
3					
4					
5					
6					
7					
8					
9					
10					

Supplementary Figure 17: Consensus-suggested changes to observation frequency for a given MEWS
