# Supplementary material for: Development of a national maternity early warning score: centile based score development and Delphi informed escalation pathways
Source: BMJ Med. 2024 May 15;3(1):e000748. doi: 10.1136/bmjmed-2023-000748 (PMC11097818; doi:10.1136/bmjmed-2023-000748)
Supplement: Supplementary data [file bmjmed-2023-000748supp002.pdf]

STROBE Statement—checklist of items that should be included in reports of observational studies

	Item No.	Recommendation	Page No.	Relevant text from manuscript
**Title and abstract**	1	(*a*) Indicate the study's design with a commonly used term in the title or the abstract	1,2	*We used measurements from the 4P prospective cohort study to estimate distributions of individual vital signs.*
		(*b*) Provide in the abstract an informative and balanced summary of what was done and what was found	2	*In a healthy population, the new MEWS alerts (at a threshold 2 or more) for 18% of observation sets, less frequently than both the Irish and Scottish maternal early warning scores (61% and 54% respectively). The centile-based score derivation approach meant each vital sign component in the new MEWS had a similar alert rate. The new MEWS had an even distribution of healthy population alerts across the antenatal period, whereas alerts increased in the third trimester with the two other systems.*
**Introduction**				
Background/rationale	2	Explain the scientific background and rationale for the investigation being reported	4	*Whole page*

Objectives	3	State specific objectives, including any prespecified hypotheses	4	*The primary objective of this study was to develop a database of vital sign measurements from pregnancy, labour and the postpartum period from which estimates of population distributions and associated centiles could be derived. The secondary objective was to use this information to develop a centile-based EWS system [9]. We have previously shown how early warning scores can be derived from distributions of vital signs [10].*
**Methods**				
Study design	4	Present key elements of study design early in the paper	5-7	*This was a mixed-methods study where we used multi-centre, observational data from the 4P study to derive a new MEWS and a Delphi process to design a consensus-derived escalation protocol.*
Setting	5	Describe the setting, locations, and relevant dates, including periods of recruitment, exposure, follow-up, and data collection	5	*Women were prospectively recruited from three UK sites*

				during the period August 2012 to December 2015.
Participants	6	(*a*) *Cohort study*—Give the eligibility criteria, and the sources and methods of selection of participants. Describe methods of follow-up<br>*Case-control study*—Give the eligibility criteria, and the sources and methods of case ascertainment and control selection. Give the rationale for the choice of cases and controls<br>*Cross-sectional study*—Give the eligibility criteria, and the sources and methods of selection of participants	5	*We used vital signs collected in the 4P study from the antenatal and two weeks post-partum period to derive a new MEWS. 4P was a longitudinal cohort study where pregnant women were approached for recruitment before 20 weeks of pregnancy at four UK maternity centres. We included women aged 16 years or over, with a singleton pregnancy, and within category one of the American Society of Anesthesiologists' classification of physical status before pregnancy ("a normal healthy patient without any clinically important comorbidity and without clinically significant past or present medical history"). Women were prospectively recruited from three UK sites during the period August 2012 to December 2015. Full details are described elsewhere*

				[8,17].
		(*b*) *Cohort study*—For matched studies, give matching criteria and number of exposed and unexposed<br>*Case-control study*—For matched studies, give matching criteria and the number of controls per case	N/A	
Variables	7	Clearly define all outcomes, exposures, predictors, potential confounders, and effect modifiers. Give diagnostic criteria, if applicable	5	*We collected the following vital signs at clinic visits every 4-6 weeks: blood pressure, heart rate, SpO2, temperature, and respiratory rate. Further details of our standard operating procedure and measurement equipment are described elsewhere [9]. We also collected demographic information (age, height, weight, self-reported ethnicity, number of previous pregnancies, smoking status), past medical and obstetric history (from participants' notes), current health status, pregnancy- related health and current medications at the initial assessment.*
Data sources/ measurement	8*	For each variable of interest, give sources of data and details of methods of assessment (measurement). Describe comparability of assessment methods if there is more than one group	5	*We collected the following vital signs at clinic visits every 4-6 weeks: blood*

				pressure, heart rate, SpO2, temperature, and respiratory rate. Further details of our standard operating procedure and measurement equipment are described elsewhere [9]. We also collected demographic information (age, height, weight, self-reported ethnicity, number of previous pregnancies, smoking status), past medical and obstetric history (from participants' notes), current health status, pregnancy- related health and current medications at the initial assessment.
Bias	9	Describe any efforts to address potential sources of bias	N/A	
Study size	10	Explain how the study size was arrived at	5	*A priori sample size calculations are described in previous publications [8,9]. In brief, to create an evidence-based early warning score we desired a 95% Confidence Interval (CI) with an standard error (SE) of less than 0.10*SD at the boundaries. We estimated a sample size of 1000 women would achieve an SE of*

			*0.05*SD at the 2.5th and 97.5th centiles, and even greater precision at the less extreme centiles. Adequate precision was also met for any subgroup analysis; for example, we estimated a sample size of 300 women would achieve an SE of 0.1*SD at the 2.5th and 97.5th centiles.*

Continued on next page

Quantitative variables	11	Explain how quantitative variables were handled in the analyses. If applicable, describe which groupings were chosen and why	5-6	*We used previously calculated models of vital signs distributions from the ante- and post-partum periods [8,18]. These distributions were fitted using Generalized Additive Models for Location, Scale and Shape [19], exploring different statistical methods to achieve the best fit to the data [8,18]. Goodness of fit was assessed by inspecting empirical centiles versus fitted centiles, quantile-quantile plots of the residuals, plots of residuals versus fitted values, and the distribution of fitted Z scores across days since birth.*
Statistical methods	12	(*a*) Describe all statistical methods, including those used to control for confounding	5-6	*We used previously calculated models of vital signs distributions from the ante- and post-partum periods [8,18]. These distributions were fitted using Generalized Additive Models for Location, Scale and Shape [19], exploring different statistical methods to achieve the best fit to the data [8,18]. Goodness of fit was assessed by inspecting empirical centiles versus fitted centiles, quantile-*

				quantile plots of the residuals, plots of residuals versus fitted values, and the distribution of fitted Z scores across days since birth.
		(*b*) Describe any methods used to examine subgroups and interactions	N/A	
		(*c*) Explain how missing data were addressed	6	*We only used observation sets where all vital signs were recorded simultaneously (complete case analysis).*
		(*d*) *Cohort study*—If applicable, explain how loss to follow-up was addressed<br>*Case-control study*—If applicable, explain how matching of cases and controls was addressed<br>*Cross-sectional study*—If applicable, describe analytical methods taking account of sampling strategy	N/A	
		(*e*) Describe any sensitivity analyses	N/A	
**Results**				
Participants	13*	(a) Report numbers of individuals at each stage of study—eg numbers potentially eligible, examined for eligibility, confirmed eligible, included in the study, completing follow-up, and analysed	8, Supplement page 4	*Table 1, supplementary figure 4*
		(b) Give reasons for non-participation at each stage	Supplement page 4	*Supplementary figure 4*
		(c) Consider use of a flow diagram	Supplement page 4	*Supplementary figure 4*
Descriptive data	14*	(a) Give characteristics of study participants (eg demographic, clinical, social) and information on exposures and potential confounders	8	*Table 1*
		(b) Indicate number of participants with missing data for each variable of interest	N/A	
		(c) *Cohort study*—Summarise follow-up time (eg, average and total amount)	N/A	
Outcome data	15*	*Cohort study*—Report numbers of outcome events or summary measures over time	*N/A*	
		Case-control study—Report numbers in each exposure category, or summary measures of exposure	*N/A*	
		Cross-sectional study—Report numbers of outcome events or summary measures	*N/A*	

Gerry S, *et al. BMJMED* 2024; 3:e000748. doi: 10.1136/bmjmed-2023-000748

Main results	16	(*a*) Give unadjusted estimates and, if applicable, confounder-adjusted estimates and their precision (eg, 95% confidence interval). Make clear which confounders were adjusted for and why they were included	N/A	
		(*b*) Report category boundaries when continuous variables were categorized	9	*Table 2*
		(*c*) If relevant, consider translating estimates of relative risk into absolute risk for a meaningful time period	N/A	

Continued on next page

Other analyses	17	Report other analyses done—eg analyses of subgroups and interactions, and sensitivity analyses	9-11	
**Discussion**				
Key results	18	Summarise key results with reference to study objectives	12	*Our new MEWS has been developed using data from a prospective observational study. The MEWS scoring thresholds were calculated based on the modelled distributions of each vital sign during the antenatal and immediate postnatal periods. We ensured a clinically appropriate and acceptable triggering rate by using centiles to determine the threshold values. We determined appropriate escalation responses to MEWS values through a Delphi process involving multi-disciplinary expert stakeholders.*
Limitations	19	Discuss limitations of the study, taking into account sources of potential bias or imprecision. Discuss both direction and magnitude of any potential bias	13	*Some limitations should be considered when evaluating the new MEWS. Firstly, the tool has not been validated against outcomes, for which a very large dataset will be required. Furthermore, the performance assessment was based on the same data used to develop score which may result in an*

				optimistic assessment of the equal weighting of each vital sign. However, the large multicentre nature of the data should however ensure generalisability and we would expect similar results in external data. Although we propose adjustment to the heart rates used to alert to deterioration in the early post-partum period, the possibility that further improvements in detection could be obtained by considering variations in other vital signs through pregnancy needs to be explored. Despite these limitations we believe we present an approach to developing an evidence-based alerting system and response pathway that moves forward the field.
Interpretation	20	Give a cautious overall interpretation of results considering objectives, limitations, multiplicity of analyses, results from similar studies, and other relevant evidence	12-13	*Whole discussion*
Generalisability	21	Discuss the generalisability (external validity) of the study results	13	*However, the large multicentre nature of the data should however ensure generalisability and we would expect similar results in external data.*

**Other information**				
Funding	22	Give the source of funding and the role of the funders for the present study and, if applicable, for the original study on which the present article is based	14	*The 4P study was funded* by the National Institute for Health Research (NIHR) Oxford Biomedical Research Centre (BRC). The views expressed are those of the authors and not necessarily those of the NHS, the NIHR or the Department of Health.

*Give information separately for cases and controls in case-control studies and, if applicable, for exposed and unexposed groups in cohort and cross-sectional studies.

Note: An Explanation and Elaboration article discusses each checklist item and gives methodological background and published examples of transparent reporting. The STROBE checklist is best used in conjunction with this article (freely available on the Web sites of PLoS Medicine at http://www.plosmedicine.org/, Annals of Internal Medicine at http://www.annals.org/, and Epidemiology at http://www.epidem.com/). Information on the STROBE Initiative is available at www.strobe-statement.org.
